# Spectral weight reduction of two-dimensional electron gases at oxide surfaces across the ferroelectric transition

**DOI:** 10.1038/s41598-020-73657-1

**Published:** 2020-10-08

**Authors:** P. Jaiban, M.-H. Lu, T. Eknapakul, S. Chaiyachad, S. H. Yao, N. Pisitpipathsin, M. Unruan, S. Siriroj, R.-H. He, S.-K. Mo, A. Watcharapasorn, R. Yimnirun, Y. Tokura, Z.-X. Shen, H. Y. Hwang, S. Maensiri, W. Meevasana

**Affiliations:** 1 School of Physics, Suranaree University of Technology, Nakhon Ratchasima, 30000 Thailand; 2 Faculty of Science, Energy and Environment, King Mongkut’s University of Technology North Bangkok, Rayong Campus, Rayong, 21120 Thailand; 3 College of Engineering and Applied Sciences and National Laboratory of Solid State Microstructures, Nanjing University, Nanjing, 210093 China; 4 Department of Applied Physics, Faculty of Science and Liberal Arts, Rajamangala University of Technology Isan, Nakhon Ratchasima, 30000 Thailand; 5 Key Laboratory of Quantum Materials of Zhejiang Province, School of Science, Westlake University, Hangzhou, 310024 Zhejiang China; 6 Advanced Light Source, Lawrence Berkeley National Lab, Berkeley, CA 94720 USA; 7 Department of Physics and Materials Science, Faculty of Science, Chiang Mai University, Chiang Mai, 50200 Thailand; 8 Center of Excellence in Materials Science and Technology, Materials Science Research Center, Faculty of Science, Chiang Mai University, Chiang Mai, 50200 Thailand; 9 School of Energy Science and Engineering, Vidyasirimedhi Institute of Science and Technology (VISTEC), Wangchan Valley, Rayong, 21210 Thailand; 10 Department of Applied Physics, University of Tokyo, Bunkyo-ku, Tokyo, 113-8656 Japan; 11 Departments of Physics and Applied Physics, Stanford University, Stanford, CA 94305 USA; 12 SIMES, SLAC National Accelerator Laboratory, 2575 Sand Hill Road, Menlo Park, CA 94025 USA; 13 Center of Excellence on Advanced Functional Materials, Suranaree University of Technology, Nakhon Ratchasima, 30000 Thailand; 14 Thailand Center of Excellence in Physics (ThEP), MHESI, Bangkok, 10400 Thailand

**Keywords:** Electronic properties and materials, Ferroelectrics and multiferroics, Phase transitions and critical phenomena

## Abstract

The discovery of a two-dimensional electron gas (2DEG) at the $\hbox {LaAlO}_3/\hbox {SrTiO}_3$ interface has set a new platform for all-oxide electronics which could potentially exhibit the interplay among charge, spin, orbital, superconductivity, ferromagnetism and ferroelectricity. In this work, by using angle-resolved photoemission spectroscopy and conductivity measurement, we found the reduction of 2DEGs and the changes of the conductivity nature of some ferroelectric oxides including insulating Nb-lightly-substituted $\hbox {KTaO}_3$, $\hbox {BaTiO}_3$ (BTO) and (Ca,Zr)-doped BTO across paraelectric-ferroelectric transition. We propose that these behaviours could be due to the increase of space-charge screening potential at the 2DEG/ferroelectric regions which is a result of the realignment of ferroelectric polarisation upon light irradiation. This finding suggests an opportunity for controlling the 2DEG at a bare oxide surface (instead of interfacial system) by using both light and ferroelectricity.

## Introduction

Since the discovery of a two-dimensional electron gas (2DEG) at the interface between the insulating oxides $\hbox {LaAlO}_3$ and $\hbox {SrTiO}_3$^1^, 2DEGs at other interfaces/surfaces of transition-metal oxides, i. e. $\hbox {LaTiO}_3$/$\hbox {KTaO}_3$ ^2^ and amorphous/crystalline oxide interfaces ^3^, have been demonstrated to exhibit a collection of novel properties, prompting applications in future multifunctional electronic devices^4,5^. The appealing properties include superconductivity^6,7^, magnetic orders^8–10^, enhanced Seebeck coefficient^11^, large negative electron compressibility^12^ and ferroelectric polarisation switching^13^. From our previous study, by using angle-resolved photoemission spectroscopy(ARPES), we showed that a similar 2DEG can be formed on the bare $\hbox {SrTiO}_3$ surface under exposure to intense ultraviolet irradiation^14^. The carrier densities were up to the same order as in the interfacial systems, and could be controlled by the UV irradiation dose which induces oxygen vacancies at the surface^15–17^. The corresponding changes of these carrier densities could also be observed from the surface resistivity^17^. Besides $\hbox {SrTiO}_3$ measurements, our extended study found that a 2DEG can also be created on $\hbox {KTaO}_3$ surfaces using the same methodology as for $\hbox {SrTiO}_3$^18^.

While the 2DEG states at both $\hbox {SrTiO}_3$ and $\hbox {KTaO}_3$ surfaces have many similar features, there is a clear difference in the 2DEG formation. At the non-polar surface of $\hbox {SrTiO}_3$, the 2DEG was absent right after cleaving and then started to form upon UV irradiation; however, at the polar surface of $\hbox {KTaO}_3$, the 2DEG could be found immediately after cleaving^18^. So, the electrostatic nature of surface can certainly influence the 2DEG formation. Indeed, there are already studies showing that external stimuli (e.g. electric field^19,20^ and UV irradiation doses^14–16^) can vary the 2DEG electron density, suggesting all-oxide-device applications and fabrication methods. There were also theoretical predictions that 2DEG states, which are formed at the interface between a ferroelectric oxide and $\hbox {SrTiO}_3$, can be controlled via ferroelectric polarisation^21,22^; experimentally, the control of 2DEG conductivity by using ferroelectric polarisation was observed in the modified structure of ferroelectric Pb($\hbox {Zr}_{0.2}\,\hbox {Ti}_{0.8}$)$\hbox {O}_3$/$\hbox {LaAlO}_3$/$\hbox {SrTiO}_3$ ^13^ and $\hbox {LaAlO}_3$/$\hbox {Ba}_{0.2}\,\hbox {Sr}_{0.8}\,\hbox {TiO}_3$ ^23^ and the modified surface of $\hbox {SrTiO}_3$  ^24,25^.

In this paper, instead of studying the interfacial system mentioned above, we are interested in studying the effect of ferroelectricity on the 2DEG state at the bare surface of a single oxide. Without interface, it is suitable for ARPES measurement which can directly measure the electronic structure of the 2DEG. Insulating lightly-substituted K(Ta,Nb)$\hbox {O}_3$ (KTN)^26^ samples are our choices for the ARPES measurement since they can host the surface 2DEG and also exhibit ferroelectricity which allowed us to observe any changes across transition temperature ($T_c$). Furthermore, we also performed irradiation-induced conductivity measurement on a number of other ferroelectric oxide samples with various $T_c$ which allow us to deduce a picture consistent with the ARPES data.

## Methods

### Sample preparation

Our samples measured in the work include both paraelectric and ferroelectric (poly)crystals. $$\hbox {SrTiO}_3$$ (STO) (Crystal Base Co., Japan) and lightly electron-doped $$\hbox {K}_{1-x}\,\hbox {Ba}_x\,\hbox {TaO}_3$$ (flux-grown samples, $$x < 0.001$$) samples are single crystals with (001) crystal orientation, representing the normal-state ones. Ferroelectric samples with various transition temperatures are $$\hbox {KNb}_{{x}}\,\hbox {Ta}_{1-x}\,\hbox {O}_3$$ (KTN) (x = 0.02, 0.03 and 0.05) with $$T_c$$$$\approx $$ 20–90 K estimated from Ref.^26^, $$\hbox {BaTiO}_3$$ (BTO) with $$T_c = 393$$ K^27^, $$\hbox {Ba}_{0.85}\,\hbox {Ca}_{0.15}\,\hbox {Zr}_{0.1}\,\hbox {Ti}_{0.9}\,\hbox {O}_3$$ (BCZT) with $$T_c = 377$$ K and ($$\hbox {Ba}_{0.7}\,\hbox {Ca}_{0.3}$$)$$_{1-1.5x}\,\hbox {La}_x\,\hbox {TiO}_3$$ (BCLT) with $$T_c$$ from 340 to 383 K^28^. BTO is a single crystal from MTI Corp., USA. KTN samples are flux-grown single crystals (for preparation method, see Ref.^29^). BCLT with *x* = 0, 0.005, 0.01, 0.03 and BCZT are polycrystals prepared by solid state reaction method; for the growth method, see the supplementary information.

### ARPES measurements

ARPES measurements (*T* = 10–160 K, $h\nu$ = 45–85 eV) of in-situ cleaved single-crystal samples were performed using a Scienta R4000 hemispherical analyser at beamline 10.0.1 of the Advanced Light Source with an energy resolution between 8 and 35 meV, and an angular resolution of $0.35^\circ$.

### Conductivity measurement

The conductivity measurement under synchrotron light was performed *in situ* at room temperature and a base pressure of $$1.4 \times 10^{-8}$$ torr (Synchrotron Light Research Institute, BL 3.2a). The measurements of irradiation-induced conductivity at the ferroelectric-sample surfaces were performed using a sourcemeter (Agilent B2901A) and a violet (405nm) laser with intensity $$\approx $$$$0.3 \,\hbox {W}/\hbox {cm}^2$$; the exposure to the violet laser is in between two gold electrodes 2 mm apart (see Fig. 3a).

## Results and discussion

**Figure 1:**
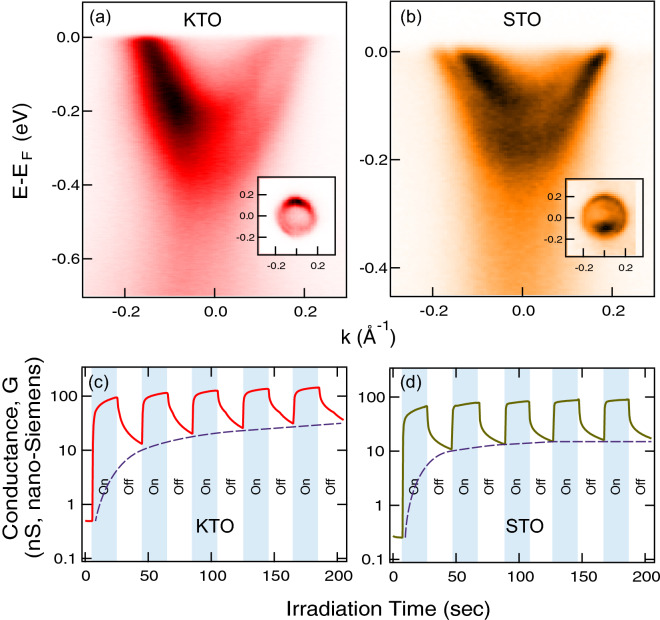
Irradiation-induced 2DEG states at the surfaces of (**a**) Ba-lightly-doped $\hbox {KTaO}_3$ (from Ref.^18^) and (**b**) La-lightly-doped $\hbox {SrTiO}_3$ (from Ref.^14^). The change of surface conductance of (**c**) $\hbox {KTaO}_3$ and (**d**) $\hbox {SrTiO}_3$ under synchrotron light irradiation measured in this work at based pressure of $1.4 \,\times \, 10^{-8}$ torr; dash lines connect the end points in the off state.

Figure 1a,b show the ARPES measurement of the normal-state undoped $$\hbox {KTaO}_3$$ and $$\hbox {SrTiO}_3$$ respectively where the insets show the corresponding Fermi surfaces. The surface carrier densities of KTO and STO, estimated from the Fermi surface area (e.g. $$n_{2D} = k_F^2/2\pi $$ for circular shape), are both in the order of $$1 \times 10 ^{14}$$$$\hbox {cm}^{-2}$$. These ARPES data indicate that the 2DEG states can be well formed on the surfaces of nearly insulating bulk crystals. The formation of 2DEG states can also be correspondingly observed from the surface conductance measurement upon intense irradiation as depicted in Fig. 1c,d. Upon increasing the exposure time, the conductances in the off states, whose contribution mostly come from the slow-changing 2DEG states^17^, increase along the dash lines, quantitatively agreeing with the trends of the increases in surface carrier densities observed in ARPES data^14,18^. By using these same ARPES and conductance measurements, we then performed further experiments on the ferroelectric samples to observe any change across their transition temperatures.

**Figure 2:**
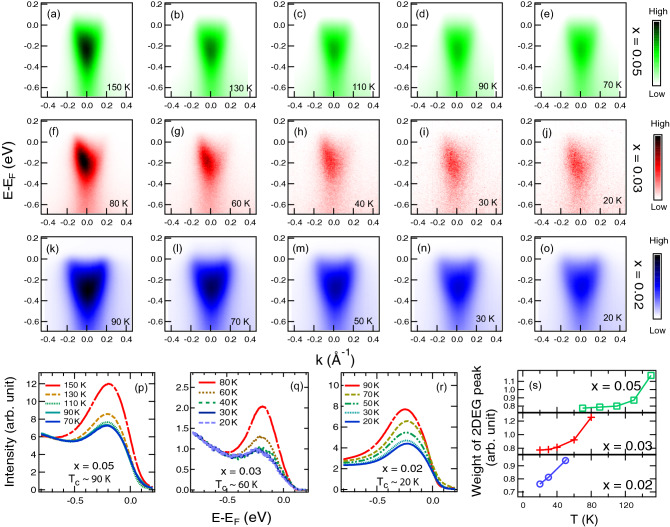
ARPES data of 2DEG states at the surfaces of $\hbox {KNb}_{{x}}\,\hbox {Ta}_{1-x}\,\hbox {O}_3$: (**a**–**e**) for x = 0.05 ($T_c\sim 90K$), (**f**–**j**) for x = 0.03 ($T_c\sim 60K$), and (**k**–**o**) for x = 0.02 ($T_c\sim 20K$) with measurement temperature as indicated in each panel. (**p**–**r**) summarise the angle-integrated photoemission intensity at each temperature for KTN with x = 0.05, 0.03 and 0.02 respectively. (**s**) shows the weight of the 2DEG peak (i.e. area under the graph) of panels (**p**–**r**) as a function of temperature; note that the intensity is normalised by the background.

To study the effect of ferroelectricity on the 2DEG formation, we firstly performed the ARPES measurement on $$\hbox {KNb}_{0.36}\,\hbox {Ta}_{0.64}\,\hbox {O}_3$$ with ferroelectric $$T_c$$$$\sim $$ 300 K. After cleaving many of these samples in vacuum at measurement temperature of 20 K, no 2DEG was observed even after applying intense irradiation for hours. The contrast between this $$\hbox {KNb}_{0.36}\,\hbox {Ta}_{0.64}\,\hbox {O}_3$$ and Ba-lightly-doped $$\hbox {KTaO}_3$$ (Fig. 1a) already suggested some effect of ferroelectricity to be investigated further in other ferroelectric samples. Unfortunately, since the $$\hbox {KNb}_{0.36}\,\hbox {Ta}_{0.64}\,\hbox {O}_3$$ has high $$T_c$$, we could not perform a reliable ARPES measurement (due to strong thermal smearing at high temperature) across the ferroelectric transition. We then chose to perform ARPES measurements on KTN samples with x = 0.02, 0.03 and 0.05, and $$T_c$$$$\approx $$ 20, 60, and 90 K respectively. As shown in Fig. 2, the conduction pockets were found in all KTN samples. These pockets are referred to as the 2DEGs formed at the polar surface of pure and Ba-lightly-doped KTO confirmed by previous photon energy dependence measurements ^18^. Here, we could well observe the 2DEG states of the KTN samples at high temperatures (relative to $$T_c$$). Then the ARPES intensity drops upon lowering the temperature. As shown in Fig. 2p–r, these changes can be well observed in the angle-integrated intensities. From these spectra in panels (p–r), the areas under the graph (i.e. proportional to the 2DEG density) as a function of temperature are summarised in Fig. 2s; this reveals an onset behaviour of the 2DEG formation near the transition temperature of each sample. Besides the ARPES intensity, there also appears that the spectral line shapes become slightly broader at lower temperature; this is in contrast to other conductive oxides where features usually become sharper at lower temperature^30^, suggesting that the change near the transition is intrinsic.

**Figure 3:**
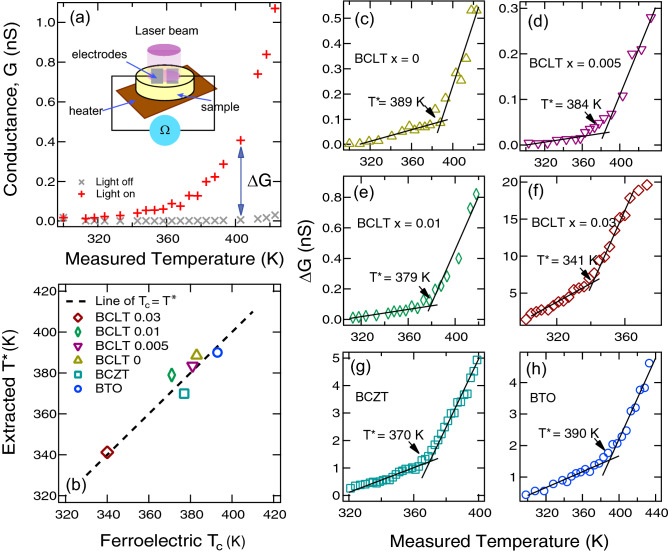
The change of conductance at the surface of ferroelectric oxides under irradiation as a function of temperature. (**a**) shows an example of surface conductance with laser light on and off where $\Delta G$ is the difference in conductance between on and off states indicated; the conductance of on and off states are shown in supplementary information. Panels (**c**–**h**) show the measured $\Delta G$ of each indicated sample as a function of temperature in the range covering the ferroelectric $T_c$; each marked $T^{*}$ indicates the temperature where the conductance trend changes its slope. Panel (**b**) summarised the extracted $T^{*}$ for each sample as a function of its ferroelectric $T_c$.

To look further into this change near the transition, we also study the temperature-dependent surface conductivity across ferroelectric transition. As shown in the diagram of Fig. 3a, we applied UV irradiation on various Ba-based titanates with ferroelectric $T_c$ between 340 - 390 K and then measured the increase in conductance ($\Delta G$) as a function of temperature. This UV exposure is for the same purpose for creating 2DEG on $\hbox {SrTiO}_3$ where its dynamics observed from ARPES and conductivity measurements were found to correspond well with each other^14,17^. As shown in Fig. 3c–h, the increases in conductance ($\Delta G$) in all the samples show a similar trend of having a rapid change across a characteristic temperature $T^*$. We define this $T^*$ as the temperature where the two straight lines fitted to data intersect each other. Then, we plot the extracted $T^*$ of each sample as a function of its ferroelectric $T_c$ as summarised in Fig. 3b. This line-up indicates that surface conductance induced by the UV-irradiation is largely decreased below $T_c$. This is in agreement with the ARPES measurements in Fig. 2 which show that the 2DEG states could be well developed in the paraelectric state but become suppressed across the ferroelectric transition.

With these two independent experiments, it is interesting that the spectral weight reduction of 2DEGs and the changes of conductivity upon light irradiation were occurred similarly in both single- and poly- crystalline ferroelectric oxides. In fact, the ferroelectric properties in various systems are different depending on many factors, i.e, ferroelectric self-polarisation characteristic, domain formation and surface chemistry ^31,32^. The coupling between ferroelectricity and 2DEGs has been proposed to be originated from the interfacial coupling mechanism at their space-charge region ^23,33,34^. This phenomenon usually appears in nanoscale, hence, effects of domain wall/substrate which cause some gradient on a much larger scale of microns ^35,36^ would be neglected.

Recent investigations show that 2DEG density can be modulated by controlling the ferroelectric polarisation ^13,23,33^. Combining with the previous research on ferroelectric La-doped BTO ^37^ reporting that the noneqilibrium charge carriers can be generated through UV irradiation which thus change the nature of charge distribution and local electric field in the ferroelectric materials. Hence, the spectral weight reduction of 2DEGs at ferroelectric state upon UV irradiation would be related to this mechanism. Overall, we proposed that irradiating the light on the ferroelectric state-oxide surfaces can align the ferroelectric polarisation through the excess of charge carriers which is not expected to occur in the paraelectric state. This ferroelectric realignment can then maximise the space-charge potential (i.e. formation of upward ferroelectric polarisation near the surface ^33^) which suppresses the formation of 2DEG density in our measured ferroelectric oxides below $\hbox {T}_c$.

## Conclusion

We have investigated the dynamics of 2DEG across the ferroelectric transition at the surfaces of several ferroelectric oxide materials. It is found that both electron density and conductivity are pronouncedly decreased across the transition. Regarding the origin of this reduction, we propose that the ferroelectric polarisation realignment induced by light irradiation increases the space-charge potential which suppresses the formation of 2DEG as well as the changes of conductivity in the ferroelectric state. Finally, our findings present the comprehensive study between three-coupled degrees of freedom, i.e. 2DEGs, ferroelectricity, and light. This therefore offers the new pathways for novel applications which are not limited only to the interfacial systems, i.e. light sensitive high electron mobility transistor.

## Supplementary information

Supplementary material 1
