# Supplementary material for: Spectral weight reduction of two-dimensional electron gases at oxide surfaces across the ferroelectric transition
Source: Sci Rep. 2020 Oct 8;10:16834. doi: 10.1038/s41598-020-73657-1 (PMC7545169; doi:10.1038/s41598-020-73657-1)
Supplement: Supplementary file 1 — Supplementary material 1 [file 41598_2020_73657_MOESM1_ESM.pdf]

Supplementary Information for
Spectral weight reduction of two-dimensional electron gases at oxide surfaces across the ferroelectric transition

P. Jaiban[1,2], M.-H. Lu[3], T. Eknapakul[1], S. Chaiyachad[1], S. H. Yao[3], N. Pisitpipathsin[4], M. Unruan[4], S. Siriroj[1], R.-H. He[5], S.-K. Mo[6], A. Watcharapasorn[7,8], R. Yimnirun[1,9], Y. Tokura[10], Z.-X. Shen[11,12], H. Y. Hwang[11,12], S. Maensiri[1,13], and W. Meevasana[1,13,*]

[1] School of Physics, Suranaree University of Technology, Nakhon Ratchasima, 30000, Thailand
[2] Faculty of Science, Energy and Environment, King Mongkut's University of Technology North Bangkok, Rayong Campus, Rayong 21120, Thailand
[3] College of Engineering and Applied Sciences and National Laboratory of Solid State Microstructures, Nanjing University, Nanjing 210093, China
[4] Department of Applied Physics, Faculty of Science and Liberal Arts, Rajamangala University of Technology Isan, Nakhon Ratchasima 30000, Thailand
[5] Key Laboratory of Quantum Materials of Zhejiang Province, School of Science, Westlake University, Hangzhou 310024, Zhejiang, China
[6] Advanced Light Source, Lawrence Berkeley National Lab, Berkeley, CA 94720, USA
[7] Department of Physics and Materials Science, Faculty of Science, Chiang Mai University, Chiang Mai, 50200, Thailand
[8] Center of Excellence in Materials Science and Technology, Materials Science Research Center, Faculty of Science, Chiang Mai University, Chiang Mai, 50200, Thailand
[9] School of Energy Science and Engineering, Vidyasirimedhi Institute of Science and Technology (VISTEC), Wangchan Valley, Rayong 21210 Thailand
[10] Department of Applied Physics, University of Tokyo, Bunkyo-ku, Tokyo 113-8656, Japan
[11] Departments of Physics and Applied Physics, Stanford University, CA 94305, USA
[12] SIMES, SLAC National Accelerator Laboratory, 2575 Sand Hill Road, CA 94025, USA
[13] Center of Excellence on Advanced Functional Materials, Suranaree University of Technology, Nakhon Ratchasima 30000, Thailand
*Corresponding e-mail: worawat@g.sut.ac.th

I. SAMPLE PREPARATION OF BCLT AND BCZT CERAMICS

For the BCLT-based ceramics, the starting oxides were $BaCO_3$, $CaCO_3$, TiO_2, and La_2O_3. Then, all oxide powders were mixed by ball milling in ethanol for 24 h, dried at 393 K for 24 h. The dried powders were calcined at 1,273 K for 2 h. The calcined powders were then mixed with 3 wt% PVA (polyvinyl alcohol) binders and subsequently pressed into pellets with a diameter of 15 mm using a uniaxial press with 1.5-t weight. Binder removal was carried out by heating the pellets at 773 K for 1 h. Then, these pellets were sintered at 1,623 K for 4 h of dwell time with a heating/cooling rate of 5 K/min.

For the BCZT ceramics, the starting oxides were $BaCO_3$, $CaCO_3$, TiO_2, and ZrO_2. All oxide powders were mixed by ball milling in ethanol for 24 h, dried at 393 K for 24 h. The dried powders were calcined at 1,473 K for 2 h. The calcined powders were then mixed with 3 wt% PVA (polyvinyl alcohol) binders and subsequently pressed into pellets with a diameter of 15 mm using a uniaxial press with 1.5-t weight. Binder removal was carried out by heating the pellets at 773 K for 1 h. Then, these pellets were sintered at 1,723 K for 4 h of dwell time with a heating/cooling rate of 5 K/min.

II. SURFACE CONDUCTANCE WITH LASER ON AND OFF

To study the conductance of 2DEG across ferroelectric transition, the conductance with laser light on and off are measured as a function of temperature as shown in Fig. S1. The measured samples were BCLT (x=0, 0.005, 0.01 and 0.03), BCZT and BTO samples. Then, the 2DEG conductance could be extracted from the difference between these on and off states as shown in Fig. 3 of the manuscript.

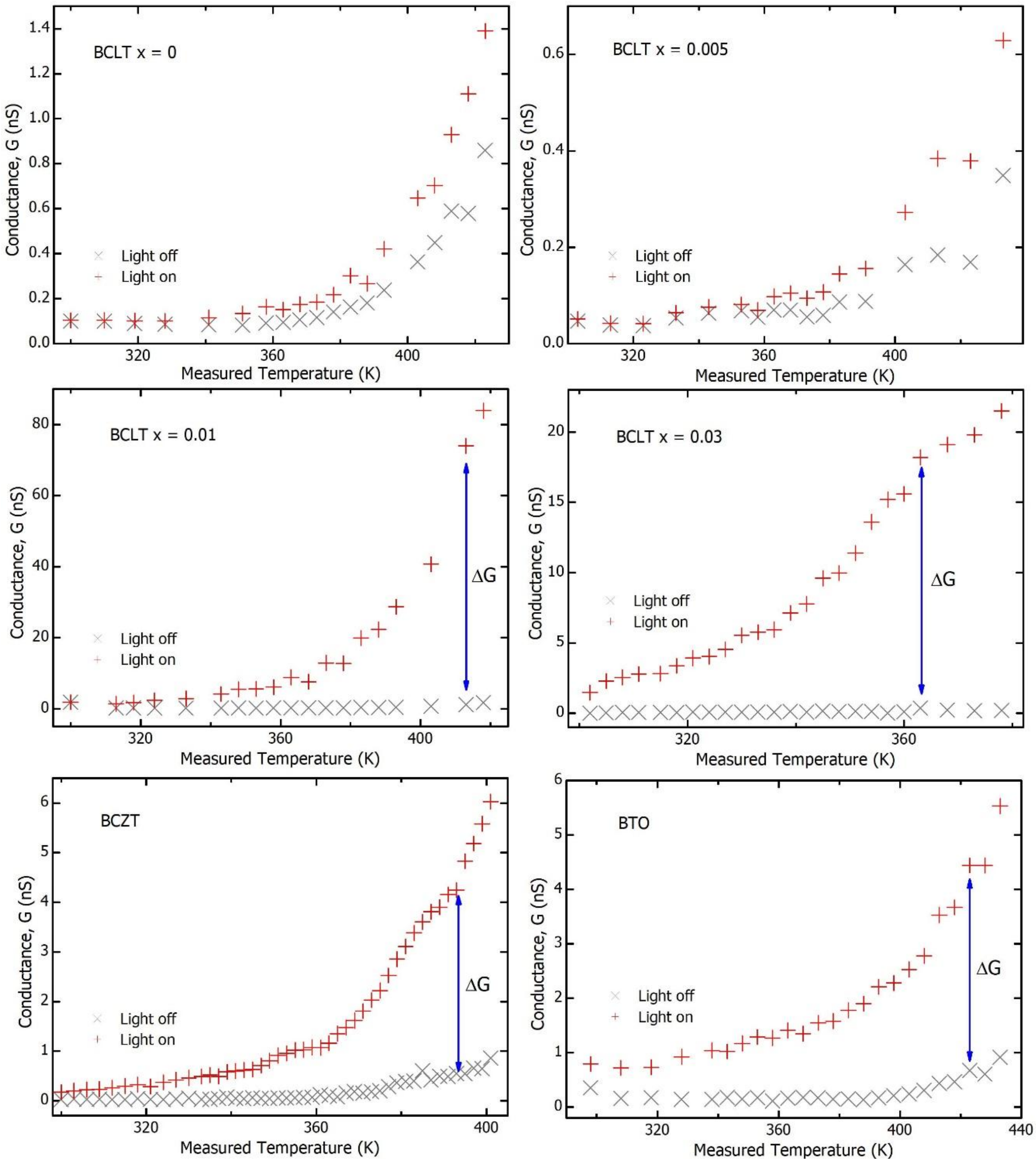

Fig. S1: Temperature-dependent conductance with laser light on and off of BCLT (x=0, 0.005, 0.01 and 0.03), BCZT and BTO samples.
